# Case report: Preputial tube-flap ureteroplasty for neoureter creation in a male dog with post ureterocolonic anastomosis complications

**DOI:** 10.3389/fvets.2024.1496603

**Published:** 2024-12-23

**Authors:** Wongsuda Yala, Anitha Chumkam, Ana Prommarit, Chanakarn Sungthong, Nut Danpanang, Wanchart Yippaditr

**Affiliations:** Kasetsart University Veterinary Teaching Hospital Hua Hin, Faculty of Veterinary Medicine, Kasetsart University, Prachuap Khiri Khan, Thailand

**Keywords:** dog, prepuce, preputial tube-flap, ureterocolonic, ureter

## Abstract

A 16.50 kg, 5-year-old male mixed breed dog presented due to complications following a ureterocolonic anastomosis performed to manage of ureteral and urinary bladder injuries. The first revision surgery involved reimplantation of the ureters into the cranial aspect of the prepuce. Dehiscence of the anastomosed ends of the right ureter was observed postoperatively. A second revision surgery was then performed, involving a preputial tube-flap ureteroplasty via preputiotomy. A longitudinal flap was raised from the prepuce and anastomosed to the end of the right ureter to create a neoureter and reduce tension at the anastomosis site. Postoperative follow-up evaluations at 10 days and 6 months revealed no unexpected complications. Six months follow-up, the owner reported that the dog exhibited a satisfactory quality of life.

## Introduction

Total cystectomy followed by ureterocolonic anastomosis was performed in 10 dogs with transitional cell carcinoma (TCC) with overall poor outcomes. Ureterocolonic anastomosis for urinary diversion has been reported as abandoned due to severe hyperammonemia, electrolyte imbalance, diarrhea, vomiting, and neurologic complications resulting from the absorption of uremic toxins across the colonic mucosa (1). More recent case reports have described reimplantation of the ureters into the cranial aspect of the vagina (2, 3), prepuce (3), and cutaneous sites (4). Although this procedure avoids the previously described complications associated with ureterocolonic anastomosis, its use must be restricted to cases in which the ureter is long enough to be reimplanted (4). A preputial tube-flap urethroplasty has been reported as a form of successful salvage surgery in the treatment of failed preputial urethrostomy (5). This technique creates additional urethral length to alleviate tension at the urethral anastomosis. The purpose of this report was to describe the use of a preputial tube-flap to create additional ureter length sufficient for reimplantation of the ureter into the cranial aspect of the prepuce, addressing postoperative complications arising from ureterocolonic anastomosis.

## Case description

**Figure 1:**
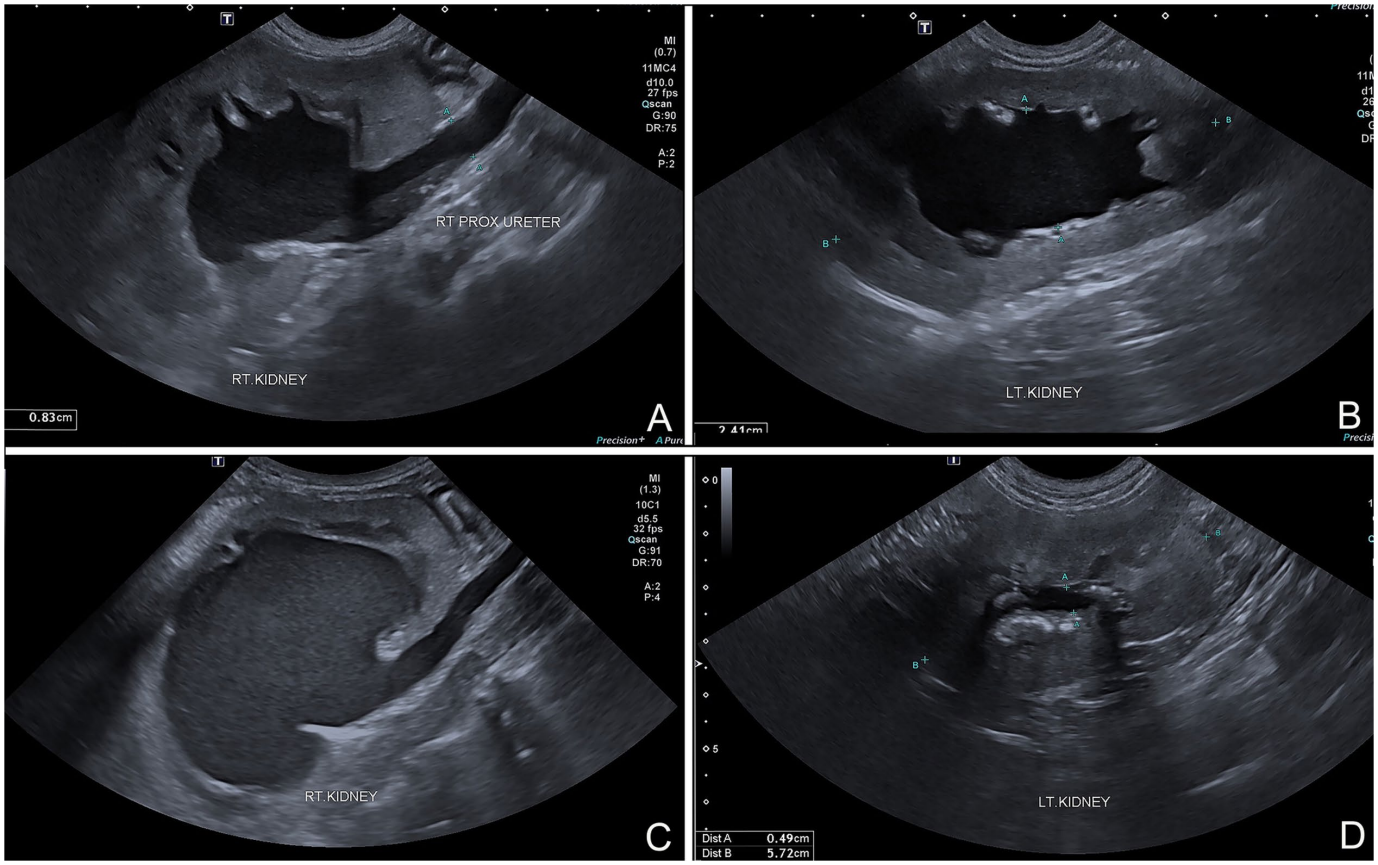
**(A)** Sagittally oriented ultrasonographic images of dog that underwent ureterocolic anastomosis. Marked hydroureteronephrosis and pyelonephritis evident in right upper urinary tract. **(B)** Hydronephrosis visible at left kidney. **(C)** Sagittal plane follow-up ultrasound images after preputial ureterostomy showing persistent and potentially worsened hydroureteronephrosis and pyelonephritis in right urinary tract. **(D)** Left kidney showing improvement in hydronephrosis, with reduced renal pelvis diameter compared to preoperative images.

**Figure 2:**
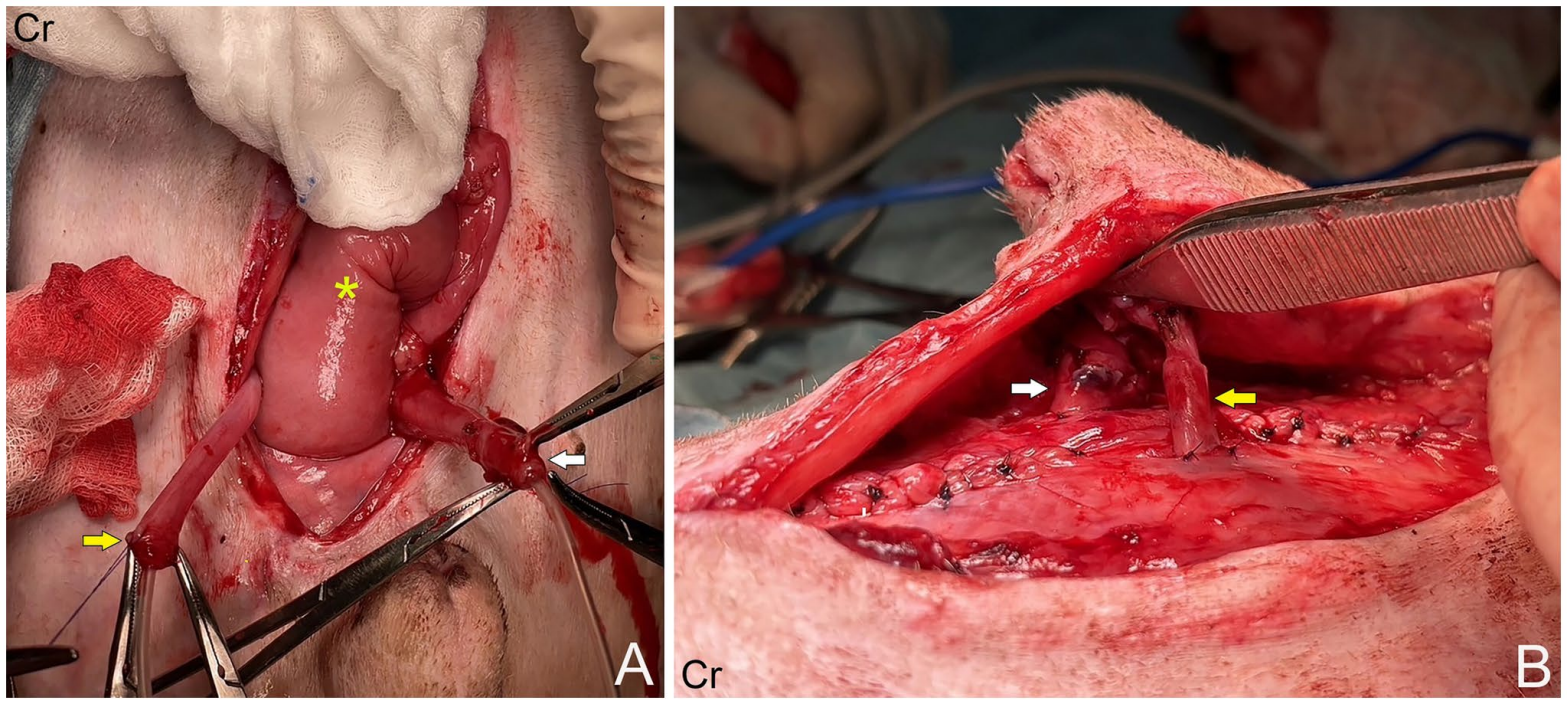
**(A)** Right ureter (yellow arrow) and left ureter (white arrow), which had been ligated and severed from the colon (asterisk). **(B)** The ureters were withdrawn through the abdominal wall into cranial aspect of prepuce.

A 16.50 kg, 5-year-old male mixed breed dog suffered from ureteric and urinary bladder injury due to complications from cryptorchid castration surgery. The first referring veterinary clinic performed total cystectomy and ureterocolonic anastomosis to address the injury. Postoperatively, the dog experienced pneumaturia and fecaluria 3–5 times daily, along with upper urinary tract infections, fever, and leukocytosis when antibiotics were discontinued. Subsequently, the dog was referred to the XXX. The rest of the clinical examination was unremarkable. A complete blood count, biochemistry panel, blood gas, and electrolyte analysis were unremarkable. The sagittal ultrasonographic kidney length-to-abdominal aortic diameter (K/AO) ratios for the left and right renal kidneys were 7.88/0.8 (9.85) and 8.24/0.8 (10.3), respectively. K/AO ratios of both kidneys exceeded the normal range of 5.5 to 9.1 observed in healthy dogs (6). Therefore, the sizes of both kidneys were significantly increased. Bilateral severe pelvicalyceal dilation and parenchymal loss detected in renal sonography (Figures 1A,B). Additionally, the right ureter exhibited dilation throughout its length and contained sediment, suggesting an upper urinary tract infection. Surgery was performed, during which the surgeon planned to reimplant the ureters into the prepuce. The procedure was conducted under general anesthesia, induced with subcutaneous (SC) morphine at 0.2 mg/kg and alfaxalone (Alfaxan; Jurox Pty. Ltd.) at 2 mg/kg intravenously (IV). Isoflurane was used for anesthesia maintenance throughout the surgery with the assistance of a circular oxygen system following endotracheal intubation. A constant-rate infusion of fentanyl (10 μg/kg/h) in normal saline was also administered throughout surgery as a pain control with rate 5 ml/kg/h. The dog was positioned in dorsal recumbency with pelvic limbs extended and abducted. The prepuce was washed with 0.12% chlorhexidine (C–20; OSOTH Inter Laboratories Co., Ltd.) as an antiseptic solution and cephalexin (Cefaben; L.B.S. Laboratory Ltd.) at 22 mg/kg IV was used for prophylactic antibiotic. The ventral midline incision was made through the caudal abdominal wall to expose the ureterocolonic anastomosis site. The ureters were ligated with 3–0 polydioxanone (PDS; Johnson & Johnson International) near the anastomosis with the colon, keeping the proximal ureters as long as possible and cutting at a macroscopically healthy site of the ureter from the colon by using Metzenbaum scissors. Urine was collected from both ureters for bacterial culture. Stay sutures with 5–0 polydioxanone (PDS; Johnson & Johnson International) were placed in the proximal ureters for manipulation during ureterostomy (Figure 2A). The abdominal wall was penetrated using haemostatic forceps with two stab incisions, and the ureters were withdrawn through the abdominal wall to the inner area of prepuce. Two orifices were created in the preputial mucosa with stab incisions. The location of these preputial mucosal orifices was determined by the residual ureteral length (in this case, they were located at the cranial aspect of the prepuce). Ureteromucosal anastomosis was performed with 5–0 polydioxanone in a simple interrupted pattern (Figure 2B). The abdominal cavity was lavaged copiously with 0.9% normal saline solution (normal saline 0.9%; G.H.P. Co., Ltd.); the laparotomy closure was routine. Postoperatively, the dog recovered well from anesthesia without any unexpected events. Marbofloxacin (Marbocyl, Vetoquinol [Thailand] Ltd.) was administered orally at 3.3 mg/kg every 24 h. Morphine (0.3 mg/kg SC) was administered every 4–6 h for 3 consecutive days and carprofen (Rimadyl; Zoetis [Thailand] Ltd.) at 4.4 mg/kg was given orally every 24 h for the following 3 days for analgesia. An Elizabethan collar was fitted to the dog immediately after recovery from anesthesia to prevent self-mutilation of the surgical site. Urine incontinence was managed with an absorbent nappy surrounding his prepuce and caudal abdomen. To prevent urine scalding, the nappy was changed every 4 h with regular cleaning and drying of his abdominal skin. Urine output was monitored every 4 h by absorbent nappy weight, showing an output 2.53 mL/kg/h. *Escherchia coli* and *Proteus mirabilis* grew on cultures from both ureters, sensitive to imipenem (Sianem, Siam [Thailand] Ltd). The dog was maintained on imipenem for 2 weeks. Fourteen days after surgery, ultrasound imaging revealed improvement in the left kidney. The renal pelvis diameter decreased, indicating improved obstruction (Figure 1D), although the corticomedullary distinction was still abnormal. Conversely, the right urinary tract exhibited marked progression of hydroureteronephrosis; the renal pelvis diameter increased and the ureter was dilated (Figure 1C), suggesting potential obstruction at the surgical site. The right kidney’s structure became poorly defined, with minimal visible renal tissue and a pelvis filled with particle-laden anechoic fluid.

**Figure 3:**
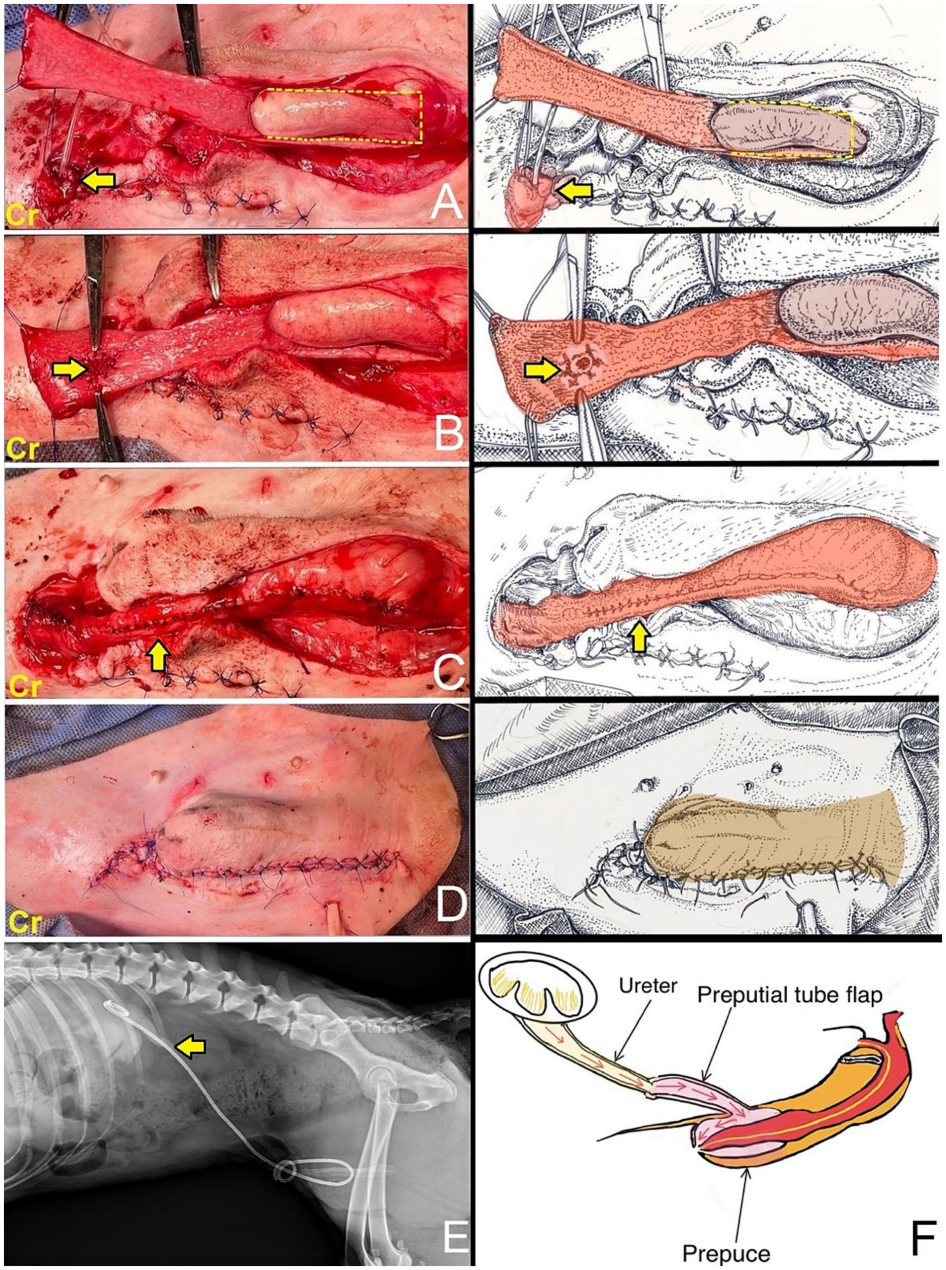
**(A)** An incision was made through the skin and subcutaneous tissue. The right ureter was adherent to the abdominal muscle wall. The indwelling catheter was inserted into the end of the previously cut ureter to identify the ureter (arrow). A full-thickness longitudinal incision was made along the lateral preputial mucosal lining toward the preputial ostium with a conjoining perpendicular incision toward the fornix, leaving the base of the flap toward the preputial orifice (dashed line). The longitudinal preputial flap was rotated to meet the end of the ureter. **(B)** A preputial mucosal tunnel was created at the cranial aspect of the flap. The ureter was pulled through tunnel and subsequently anastomosed to the preputial mucosa (arrow). **(C)** Lateral borders of the flap were sutured to create a tube. The preputial mucosa around the flap elevation site was closed (arrow). **(D)** Subcutaneous and skin layers closed. **(E)** Lateral radiograph demonstrates a ureteral catheter (arrow) positioned in the renal pelvis, with the opposite end located in the prepuce. **(F)** The diagram illustrates the flow of urine from the kidney, through the ureter, into the preputial tube flap, then into the prepuce, and finally exits through the preputial ostium.

**Figure 4:**
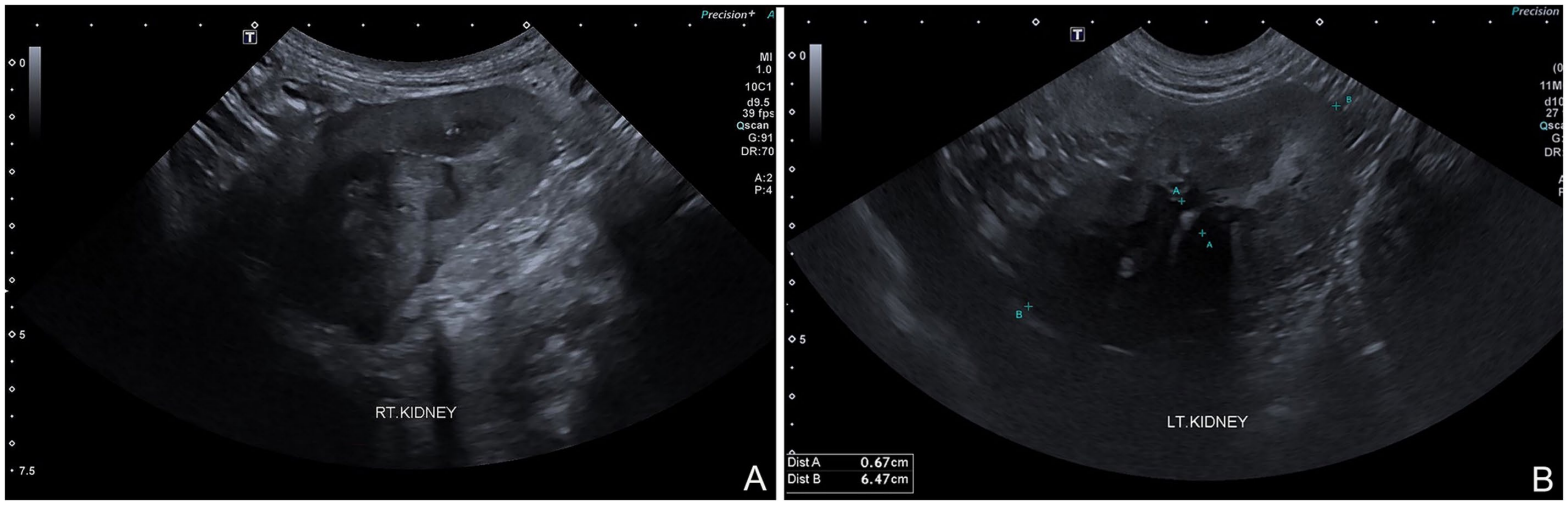
Sagittal ultrasound images following right preputial tube-flap urethroplasty. Both kidneys demonstrate residual anechoic fluid in renal pelvis, with increased echogenicity of both cortex and medulla, and poor corticomedullary junction differentiation **(A,B)**. Pyelonephritis no longer observed.

The dog was taken to revision surgery for right ureter obstruction. Before surgery, the dog underwent a complete blood count, biochemistry panel, blood gas, and electrolyte analysis. The results were unremarkable. The anesthesia protocol was the same as the previous operation. A right parapreputial incision was made through the skin and subcutaneous layers. Dehiscence of the anastomosed ends of the right ureter was observed intraoperatively. The right ureter was fibrotic and adherent to the abdominal muscle wall, 3 cm from the cranial border of the preputial orifice (Figure 3A). The ureter was identified and cut proximal to the obstruction using Metzenbaum scissors and surgical debridement was performed on the fibrotic ureter tissues. The distance between the distal end of the ureter and the prepuce was too great to allow for a tension-free anastomosis with prepuce. Therefore, a tubularized longitudinal flap was raised from the preputial mucosa to anastomose the prepuce with the end of the ureter and reduce tension at the anastomosis site. A full-thickness 5 cm longitudinal incision was made starting 1 cm distal to the fornix, along the lateral preputial mucosal lining, toward the preputial ostium. Then, a second full-thickness incision was made parallel to 1.6 cm apart from the first. A preputial mucosal flap was created with a conjoining perpendicular incision toward the fornix, leaving the base of the flap toward the preputial orifice. Then, the longitudinal preputial mucosal flap was rotated to meet the end of the ureter. A preputial mucosal tunnel was created at the cranial aspect of the flap. Stay sutures with 4–0 polydioxanone were placed to stabilize the cut end of the ureter, which was pulled through tunnel to the flap. The ureter was spatulated by making a 2–3 mm longitudinal incision in the cut end and the ureter was anastomosed to the prepuce flap using 5–0 polydioxanone with a simple interrupted pattern, ensuring that the mucosal layer of the preputial flap was facing the lumen (Figure 3B). A 6-Fr ureteral catheter with guidewire (Ureter Stent Set Paediatric; Marflow AG [Thailand] Ltd.) was inserted from the prepuce through the ureter to the kidney as a temporary stent to facilitate the reconstruction and prevent urinary irritation at the anastomosis site and tube-flap. The lateral borders of the flap were sutured around the catheter to create a tube of the preputial flap using a simple interrupted pattern with 5–0 polydioxanone (Figure 3C). The preputial mucosa around the flap elevation site was closed using a simple interrupted pattern with 4–0 polydioxanone. Finally, the subcutaneous and skin layers were lavaged with 0.9% normal saline solution. A penrose drain was placed into the subcutaneous layer. The subcutaneous tissue and skin closure were routine (Figure 3D). The catheter was maintained for 10 days (Figure 3E). Postoperatively, the dog recovered well from anesthesia without any unexpected events. The medication protocol and postoperative care managed were the same as those used in the first post-operation phase. Ten days after surgery, no subcutaneous swelling or fluid accumulation was detected during the wound examination, indicating that there was no urine leakage at the surgical site. As a result, the ureteral catheter was removed. The complete blood count, blood urea nitrogen, and creatinine levels were unremarkable. Due to the successful drainage of urine from the right kidney, ultrasound evaluation revealed substantial improvement in the right kidney (Figure 3F). The renal pelvis diameter was considerably reduced, approaching normal limits, with minimal trace of hydronephrosis (Figure 4A). However, mild hydronephrosis was still visible in the left kidney (Figure 4B). Overall, the size of both kidneys had returned to normal. However, some characteristics suggestive of chronic kidney disease were observed. Following catheter removal, the dog had urinary incontinence, with a urine output 2.83 mL/kg/h. Three days after the catheter removal, the dog was discharged from the hospital. Follow-up at approximately 6 months after surgery indicated that the complete blood count and biochemical profile were within reference ranges. Ultrasound imaging revealed mild hydronephrosis and hydroureter in both kidneys with no evidence of pyelonephritis. When asked about the dog’s quality of life, the owner considered the dog exhibited a satisfactory quality of life.

## Discussion

Historically, the use of ureterocolonic anastomosis for urinary diversion has led to many complications. Cutaneous ureterostomy and ureteral transplantation to the prepuce have been associated with fewer complications than ureterocolonic anastomosis (3). Cutaneous ureterostomy has been successfully used in dogs undergoing radical cystectomy as treatment for invasive bladder neoplasia (4, 7). However, uremic dermatitis and the need for lifelong hygiene management postoperatively must be considered with this technique (4, 7, 8). In addition, human studies have reported that body morphology, specifically obesity, is a significant factor to consider when performing ureterocutaneostomy. The anatomical changes and alterations in the vascular supply of the ureters can lead to microcirculation issues when they are stretched to the skin level during the procedure, potentially impacting surgical outcomes (9). The use has been reported of a subcutaneous ureteral bypass with a silicone catheter, which is introduced to the dorsal aspect of the penile urethra via a small urethrostomy. However, limited information is currently available regarding this approach, with the extensive implantation of foreign material in the treated dog perhaps complicating the management of urinary tract infection due to the large surface area available for biofilm formation (10). For the dog described in this report, we considered ureteral transplantation to the prepuce to treat complications from ureterocolonic anastomosis. Unfortunately, dehiscence of the anastomosed ends of the right ureter with the cranial aspect of the prepuce was observed postoperatively. The distance between the right ureter and the cranial aspect of the prepuce was too long to allow for tension-relieving ureteral transplantation (11, 12).

Various studies in humans and a veterinary case report have used preputial island tube-flap urethroplasty to create additional urethral length by forming an anastomosis between the flap and distal urethra, effectively creating a neourethra (5, 13–15). Preputial tissue is considered as an ideal donor site due to its thin, pliable membrane, watertight nature, normal functioning in a moist environment, and resistance to urine scalding (16–19). In addition, there has been a report of a modified Boari flap technique that used a tubularized bladder flap to create a tension-free ureteroneocystostomy for treating proximal ureteral obstruction in a cat (20). Based on the features and the reports on island tube-flap urethroplasty and the tubularized bladder flap, we modified this technique to create additional ureter length by forming a tubularized longitudinal flap from the prepuce to anastomose with the end of the ureter. In creating a tubularized longitudinal flap, the length of the longitudinal preputial flap should be slightly greater than the distance between the prepuce and ureter to avoid excessive tension during anastomosis, and the flap’s length-to-width ratio should not exceed 3:1 in order to preserve the blood supply to the prepuce (20–22). Postoperative complications associated with the preputial tube-flap urethroplasty and Boari flap in humans may include urethrocutaneous fistula, strictures, urinary tract infection, and urine leakage (21–23). However, in this case, the dog exhibited no signs of these complications following surgery, aside from urinary incontinence. It is important to note that incontinence is an expected outcome of preputial ureterostomy, and this should be explained to the pet owner prior surgery (3). Additionally, owners should be counseled that this procedure may be contraindicated in dogs that are intolerant to direct handling for hygiene maintenance, as described in the postoperative care section; furthermore, life-long supportive care for pyelonephritis and chronic kidney disease might be necessary (3).

Preputial tube-flap ureteroplasty can be successfully used as a salvage surgery to treat failed preputial ureterostomy. This technique creates additional ureter length that may alleviate tension at the ureter anastomosis and could also be beneficial in cases where there is insufficient ureter length for performing preputial ureterostomy, thereby mitigating tension-related complications.

## Data Availability

The original contributions presented in the study are included in the article/supplementary material, further inquiries can be directed to the corresponding author.
